# Inhibition of Quorum Sensing-Controlled Virulence Factor Production in *Pseudomonas aeruginosa* PAO1 by Ayurveda Spice Clove (*Syzygium Aromati*cum) Bud Extract

**DOI:** 10.3390/s120404016

**Published:** 2012-03-27

**Authors:** Thiba Krishnan, Wai-Fong Yin, Kok-Gan Chan

**Affiliations:** Division of Genetics and Molecular Biology, Institute of Biological Sciences, Faculty of Science, University of Malaya, Kuala Lumpur 50603, Malaysia; E-Mails: thibavengdes@yahoo.com (T.K.); yinwaifong@yahoo.com (W.F.Y.)

**Keywords:** Ayurveda, bioluminescence, biosensors, *Chromobacterium violaceum*, *lecA::lux*, *N*-acylhomoserine lactones, *Pseudomonas aeruginosa*, pyocyanin, *Syzygium aromaticum*, swarming

## Abstract

Quorum sensing controls the virulence determinants in most proteobacteria. In this work, the hexane, chloroform and methanol extracts of an Ayurveda spice, namely clove (*Syzygium aromaticum)*, shown anti-quorum sensing activity. Hexane and methanol extracts of clove inhibited the response of *C. violaceum* CV026 to exogenously supplied *N*‐hexanoylhomoserine lactone, in turn preventing violacein production. Chloroform and methanol extracts of clove significantly reduced bioluminescence production by *E. coli* [pSB1075] grown in the presence of *N*-(3-oxododecanoyl)-l-homoserine lactone. We demonstrated that clove extract inhibited quorum sensing-regulated phenotypes in *Pseudomonas aeruginosa* PA01, including expression of *lecA::lux* (by hexane extract), swarming (maximum inhibition by methanol extract), pyocyanin (maximum inhibition by hexane extract). This study shows that the presence of natural compounds that exhibit anti-quorum sensing activity in the clove extracts may be useful as the lead of anti-infective drugs.

## 1. Introduction

Bacteria communicate with each other within their vicinity using chemical signalling molecules which are known as autoinducers. Quorum sensing (QS) relies on these autoinducers to control gene expression in response to changes in bacterial cell density [1]. Several signalling molecules have been identified and the most common type in most Gram-negative bacteria are the *N*-acylhomoserine lactones (AHLs) [2]. Most recently, AHL production has been shown in an oral bacterium, namely *Klebsiella pneumoniae* [3]. At a certain threshold, where AHL concentration increases due to increased bacterial cell density, AHL will bind to its cognate receptor (luxR protein) and transcription regulation of the target genes will take place [4].

QS regulates diverse bacterial functions including antibiotic formation, virulence factor expression, luminescence, biofilm formation, motility and pigment production [5,6]. For example, *Pseudomonas aeruginosa* PAO1 is an important opportunistic pathogen, primarily infecting immunocompromised patients [7]. It uses QS to regulate several extracellular virulence factors such as LasA protease, LasB elastase, pyoverdin, pyocyanin and biofilm [8]. There are two QS systems in *P. aeruginosa* PAO1, firstly the AHL-based LasI/RhlI (which synthesize *N*-(3-oxododecanoyl)-l-homoserine lactone (3-oxo-C12-HSL) and *N*-butanoylhomoserine lactone (C4-HSL), respectively) and its regulator LasR/RhlR. Secondly, there is another QS system which employs 2-heptyl-3-hydroxy-4-quinolone (PQS) as their signal molecule for virulence factor production [9,10].

Thus, inhibiting QS, or anti-QS is an important anti-infective measure that does not rely on antibiotics [11]. Anti-QS agents will inhibit the QS mechanism and attenuate the virulence determinants and are unlikely to cause drug-resistance problems [12]. Recently, studies have found that many eukaryotes produce anti-QS compounds [13]. For example, the marine alga *Delisea pulchra* produces halogenated furanone compounds which are analogs to naturally occurring AHL signal molecules and appear to act against AHLs signal receptor proteins [14].

Originating in India, Ayurveda is known as the oldest of all traditional systems of medicine. In fact, the ancient text of Ayurveda reports more than 2,000 plant species for their therapeutic potentials [15]. Herbs and spices are common ingredients used in food preparation among the Indians because of their flavouring and preservative properties [16]. In Ayurveda, large number of herbs and spices are used as preventive and curative medicines. Screening of herbal medicinal plants for their bio-activity is important since traditional medicines are used as complementary and alternative medicine widely.

*Syzygium aromaticum* (cloves), a spice used in Ayurveda, is a source of anti-microbial agents against oral bacteria that are commonly associated with dental caries and periodontal disease [17]. It was also reported that *S. aromaticum* have been successfully used for asthma and various allergic disorders by oral administration [18]. Previous studies have shown that clove oil possesses anti-QS activities towards *C. violaceum* CV026 and swarming motility in *P. aeruginosa* PA01 [19]. Therefore, the present work studied the potential of the hexane, chloroform and methanol extract of cloves, dried flower buds of *Syzygium aromaticum* (L.) Merr. et Perry as QS inhibitor.

## 2. Experimental Section

### 2.1. Extraction

Dried clove buds were obtained from local store in Kuala Lumpur, Malaysia, in 2010. The clove buds were further dried and blended into a fine dry powder. The clove powder (50 g) was soaked separately with 250 mL of hexane, chloroform and methanol in a 500 mL conical flask for 48 hours at room temperature, without shaking. The respective solvents were filtered through Whatman filter paper No. 1 and concentrated on a rotary vacuum evaporator. Concentrated extracts were then dried in a laminar hood and were further dried in a desiccator to obtain the crude extract. The crude extract was reconstituted in dimethyl sulfoxide (DMSO) to make a stock solution of 10 mg/mL and stored at −20 °C. Further dilutions were made with ultrapure water before use. The extract was also checked for sterility by streaking on LB agar.

### 2.2. Bacterial Strains, Plasmids and Growth Conditions

Bacterial strains and plasmids used in this study are listed in Table 1. All the strains were cultured in Luria Bertani (LB) broth (1% peptone, 0.5% yeast extract, 0.5% NaCl, per 100 mL distilled water) with shaking (220 rpm). *Chromobacterium violaceum* CV026 was grown at 28 °C while all other strains were routinely cultured at 37 °C supplemented with antibiotic when necessary.

### 2.3. Anti-QS Against C. Violaceum CV026

Fifteen milliliters of warm molten LB agar were seeded overnight with *C. violaceum* CV026 culture (3 mL) grown to an OD_600_ of approximately 1.2 and *N*-hexanoylhomoserine lactone (C6-HSL, 0.15 μg/mL) as an exogenous AHL. The agar were gently mixed and poured immediately into a Petri dish. Wells (diameter of 4 mm) were made on the solidified agar plate. Sterile plant extract was loaded into the well (40 μL per well). (+)-Catechin [24] was used as positive control for QS inhibition while DMSO as negative control. The plate was incubated for 18–24 hours at 28 °C to check for the violacein inhibition. Halo zone showed anti-QS while clear and transparent zones indicated antimicrobial activity. For all instances, viability of biosensor cells was determined to rule out possibility of antibiotic effects against the biosensors.

### 2.4. Bioluminescence Assays

Bioluminescence and optical density were determined in 96-well microtitre plates using a Tecan luminometer (InfiniteM200) as previously described with slight modification [25]. Briefly, overnight cultures of *E. coli* [pSB401], *E. coli* [pSB1075] or *P. aeruginosa* PAO1 *lecA*::*lux* were diluted 1:100 in sterile, fresh LB medium, and aliquot (180 μL) with appropriate exogenous AHLs were loaded into each well containing 20 μL of sterile plant extract. For *E. coli* [pSB401] and *E. coli* [pSB1075], *N*-(3-oxohexanoyl)-l-homoserine lactone (3-oxo-C6-HSL, 0.005 μg/mL) and 3-oxo-C12-HSL (0.1 μg/mL) were supplemented, respectively. Bioluminescence and optical density were automatically simultaneously determined every 30 min for 24 hours. Bioluminescence is given as relative light units (RLU) per unit of optical density at 495 nm, which accounted for the influence of increased growth on the total bioluminescence. Experiments were done in triplicate and repeated three times.

### 2.5. Inhibition of QS-Mediated Virulence Determinants

#### 2.5.1. Swarming Assay

The swarm plates of *P. aeruginosa* PAO1 were prepared using 0.5% Bacto agar, 0.5% peptone, 0.2% yeast extract and 1.0% glucose, per 100 ml distilled water [26]. A 250 μL of sterile clove extract was seeded with 5 mL of the agar and poured immediately on a 10 mL of pre-warmed agar plate as an overlay. Two microliters of the *P. aeruginosa* PAO1 culture was inoculated at the center of the agar surface and the plate was incubated for 16 hours at 37 °C. To determine whether exogenous supplied AHLs could restore *P. aeruginosa* PAO1 swarming motility, warm swarm agar was added with C4-HSL), 3-oxo-C12-HSL or both. AHLs were applied at the concentrations of 50 μM and 100 μM.

#### 2.5.2 Pyocyanin Assay

Pyocyanin was extracted from overnight *P. aeruginosa* PAO1 culture supernatant as previously described [27] with slight modifications. Briefly, 250 μL of plant extracts were mixed with freshly prepared *P. aeruginosa* PAO1 culture (5 mL, OD_600_ = 0.1) and incubated overnight at 37 °C. Chloroform (3 mL) was added to the *P. aeruginosa* PAO1 culture supernatant and mixed vigorously. The chloroform layer was mixed with 1 mL of HCl (0.2 M). After centrifugation (10 min, 28 °C, 8,000 rpm), the OD of the HCl layer was measured at 520 nm against 0.2 M HCl using an UV/Vis spectrophotometer UV-1601 (Shimadzu, Tokyo, Japan).

### 2.6. Statistical Analysis

All the results represent the average of three independent experiments. The data were presented as mean ± standard deviation (SD) and analysed by one-way analysis of variance with P < 0.05 being significant, calculated using the GraphPad Prism 5 statistical software.

## 3. Results and Discussion

### 3.1. Clove Crude Extracts Showed Anti-QS Properties

**Figure 1.**
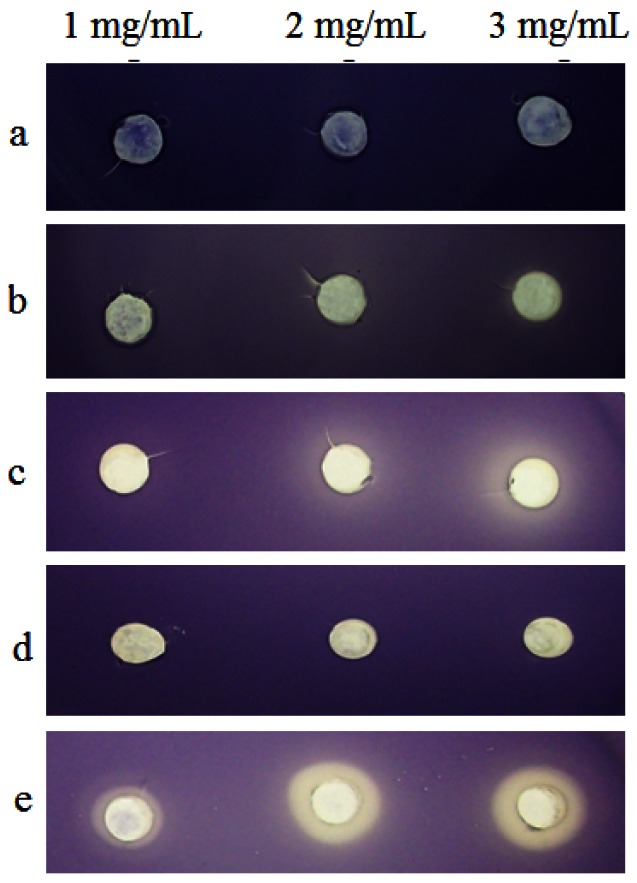
Anti-QS activity of clove crude extract against *C. violaceum* CV026. The extracts shown are: (**a**) DMSO (negative control); (**b**) (+)-catechin (positive control); (**c**) Hexane extract; (**d**) Chloroform extract; (**e**) Methanol extract; applied at 1, 2 and 3 mg/mL.

Initial screening of clove extracts for anti-QS activities were done using the *C. violaceum* CV026 as preliminary bioassay. *C. violaceum* CV026 is unable to produce the purple pigment violacein unless C6-HSL or a variety of other short-chain AHLs were supplied exogenously [20]. Increased amount of clove methanolic extract showed stronger inhibition of *C. violaceum* CV026 responding to AHL, thus inhibited its violacein production. However, hexane extract only showed observable inhibition at higher concentration of 3 mg/mL (Figure 1). Therefore, clove methanolic extract had a stronger inhibition activity than hexane extract although both extracts shown to inhibit *C. violaceum* CV026. Chloroform extract shows no activity against *C. violaceum* CV026. Different solvents, hexane (more non polar), chloroform (non polar) and methanol (polar) were used in the plant extractions because of their ability to extract various compounds due to different polarity. Consequently, different degree of anti-QS activities was observed by these extracts. As a prerequisite, we have verified that the extracts did not show bactericidal effect on all biosensor cells as determined by viability of cells (data not shown). In the present experimental condition, DMSO (at 1, 2 and 3 mg/mL) did not show significant antimicrobial effect in the performed assays when applied at these concentrations.

### 3.2. Bioluminescence Assays

#### 3.2.1. Inhibition of Luminescence Produced by *E. coli* [pSB401] and *E. coli* [pSB1075]

The hexane, chloroform and methanol extracts of clove were assayed using *lux*-based biosensor strains *E. coli* [pSB401] and *E. coli* [pSB1075] in 96-microtitre well plates. *E. coli* [pSB401] and *E. coli* [pSB1075] biosensor strains respond by the emission of light, preferentially to the presence of exogenous AHLs with acyl chains from six to eight carbons in length (such as 3-oxo-C6-HSL, C6-HSL and *N*-octanoylhomoserine lactone (C8-HSL)) or AHLs with acyl chains of 10–14 carbons in length, respectively [23].

**Figure 2.**
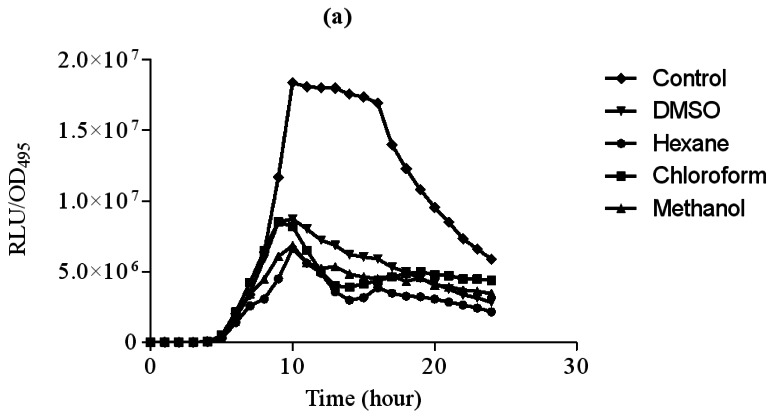

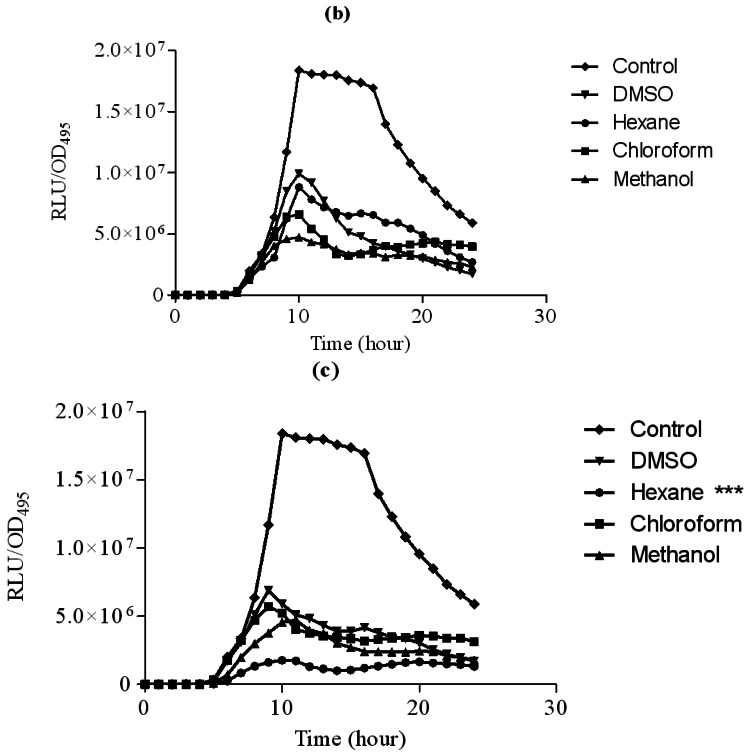
Extracts of clove inhibited bioluminescence of *E. coli* [pSB401] with increasing concentration (**a**) 1 mg/mL; (**b**) 2 mg/mL and (**c**) 3 mg/mL DMSO (inverted triangle), hexane (asterisk), chloroform (square) and methanol (triangle) extracts of clove. *E. coli* [pSB401] supplemented with 3-oxo-C6-HSL served as control (diamond) was included. The data were presented as RLU/OD to account for any differences in growth. Each point represents the mean of results from independent triplicate experiments. Data were analysed by one-way analysis of variance with *** P < 0.05 being significant.

**Figure 3.**
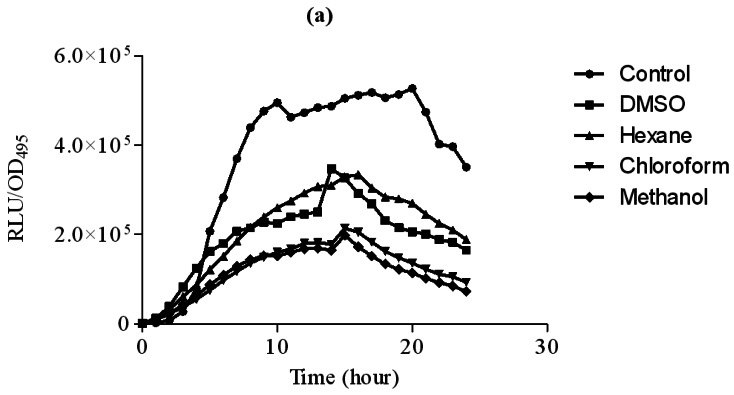

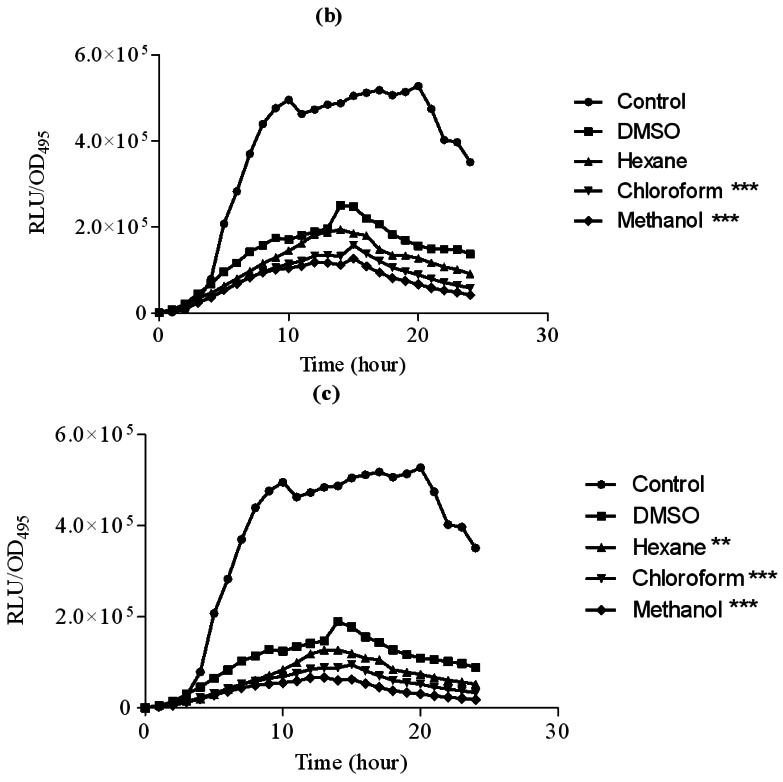
Extracts of clove inhibited bioluminescence of *E. coli* [pSB1075] with increasing concentration (**a**) 1 mg/mL; (**b**) 2 mg/mL and (**c**) 3 mg/mL of DMSO (square), hexane (triangle), chloroform (inverted triangle) and methanol (diamond) extracts of clove. *E. coli* [pSB1075] supplemented with 3-oxo-C12-HSL served as control (circle) was included. The data were presented as RLU/OD to account for any differences in growth. Each point represents the mean of results from independent triplicate cultures. The data were analysed by one-way analysis of variance with *** P < 0.05 being significant.

In this study, increasing concentration of hexane extract of clove showed significant inhibition of bioluminescence produced by *E. coli* [pSB401] biosensor strain in the presence of 3-oxo-C6-HSL compared to DMSO which served as control (Figure 2). Other extracts only showed observable bioluminescence inhibition. On the other hand, increasing concentration of chloroform and methanol significantly inhibit bioluminescence production by *E. coli* [pSB1075] strains grown in the presence of 3-oxo-C12-HSL (Figure 3). Hexane extracts of clove only showed significant inhibition at 3 mg/mL concentration compared to DMSO which served as control.

#### 3.2.2. Inhibition of P. aeruginosa PAO1 lecA::lux Activity

**Figure 4.**
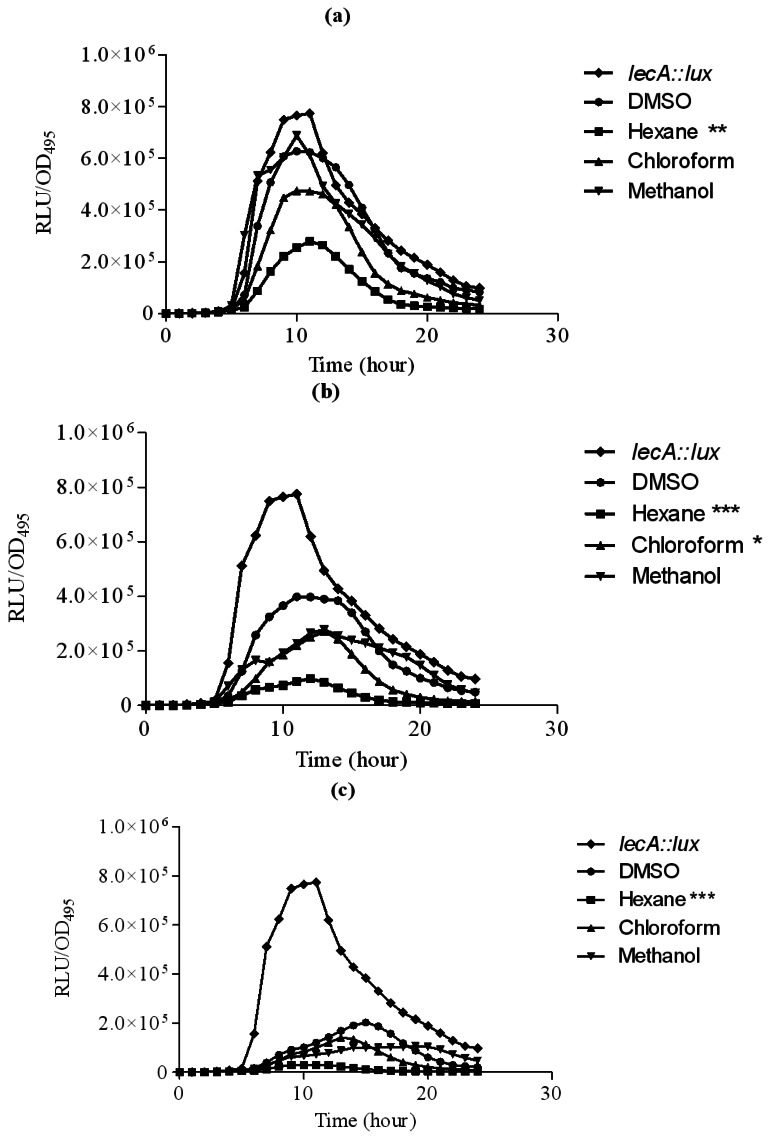
Inhibition of *P. aeruginosa* PAO1 *lecA::lux* expression with increasing concentrations of solvent or extracts added at (**a**) 1 mg/mL; (**b**) 2 mg/mL and (**c**) 3 mg/mL. Legend: *lecA::lux* (diamond), DMSO (circle), hexane (square), chloroform (triangle) and methanol extracts (inverted triangle) of clove. The data were presented as RLU/OD to account for any differences in growth. Each point represents the mean of results from independent triplicate cultures. The data were analysed by one-way analysis of variance with *** P < 0.05 being significant.

Hexane extract of clove showed significant inhibition of the *P. aeruginosa* PAO1 *lecA* expression as judged by the reduced bioluminescence of *P. aeruginosa* PAO1 *lecA::lux* fusion. However, other clove extracts only slightly inhibited the expression of the *P. aeruginosa* PA01 *lecA::lux* reporter fusion (Figure 4). This result suggests that *P. aeruginosa* PAO1 LecA virulence factor could be interrupted by the clove crude extract without affecting its growth. In these assays, all the data were analyzed using ANOVA and Dunnet's post test to compare the plant extracts (hexane, chloroform and methanol) as compared to the control (DMSO). Therefore, significant results shown are due to the effect of the plant extracts but not DMSO.

### 3.3. Inhibition of P. aeruginosa PAO1 Virulence Factors

**Figure 5.**
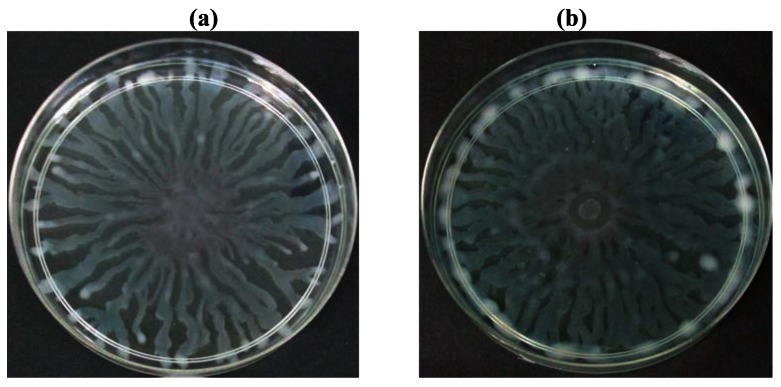

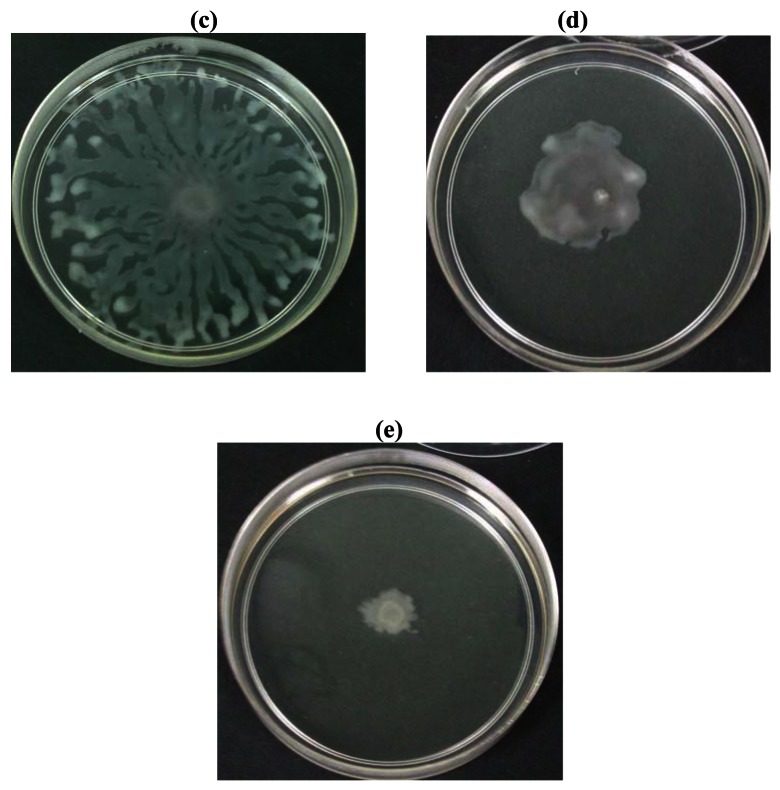
Inhibition of *P. aeruginosa* PAO1 swarming by the hexane, chloroform and methanol extracts of clove. (**a**) *P. aeruginosa* PAO1 (no addition of solvents or extracts); (**b**) DMSO; (**c**) Hexane extract; (**d**) Chloroform extract; (**e**) Methanol extract.

**Figure 6.**
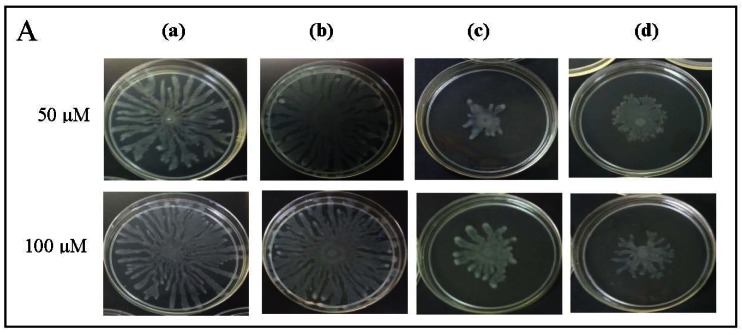

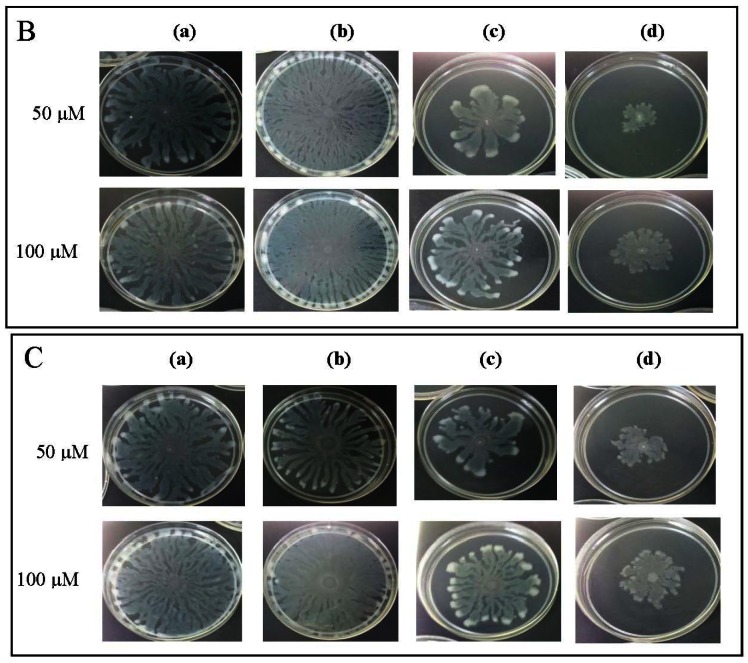
Inhibition of swarming by various extracts of clove with the addition of exogenous (**A**) C4-HSL; (**B**) 3-oxo-C12-HSL or (**C**) both (C4-HSL and 3-oxo-C12-HSL). (**a**) DMSO; (**b**) Hexane extract; (**c**) Chloroform extract; (**d**) Methanol extract.

To further explore anti-QS potential of clove extracts, further assays were done on inhibition of *P. aeruginosa* PAO1 swarming and pyocyanin production. The hexane, chloroform and methanol extracts of clove were assayed for inhibition of *P. aeruginosa* PA01 swarming motility. Maximum reduction of swarming was observed in the swarming plates containing clove methanolic extract followed by chloroform extract compared with control (DMSO) while hexane extract showed no observable reduction (Figure 5). We further verified whether *P. aeruginosa* PA01 swarming could be restored by the addition of exogenous AHLs (C4-HSL, 3-oxo-C12-HSL, and both; applied at 50 μM and 100 μM). It is clearly shown that clove methanolic extract displays consistent swarming motility inhibition regardless of the types of AHLs supplemented to the swarm agars (Figure 6). C4-HSL and 3-oxo-C12-HSL were chosen because *P. aeruginosa* PA01 employs these AHLs as its natural QS molecules to control various QS-mediated phenotypes. We further demonstrated that even when both AHLs were added at a very high concentration (100 μM), swarming of *P. aeruginosa* PA01 was not restored significantly (Figure 6). Addition of high concentration of AHLs failed to restore swarming of *P. aeruginosa* PA01 leads to the speculation that clove extracts may contain active molecules that show anti-QS activity that may not compete with AHLs to bind the cognate receptor at the same active site. This, however, requires further work on studying the interaction of clove extracts and *P. aeruginosa* PA01 luxI and luxR homologues.

**Figure 7.**
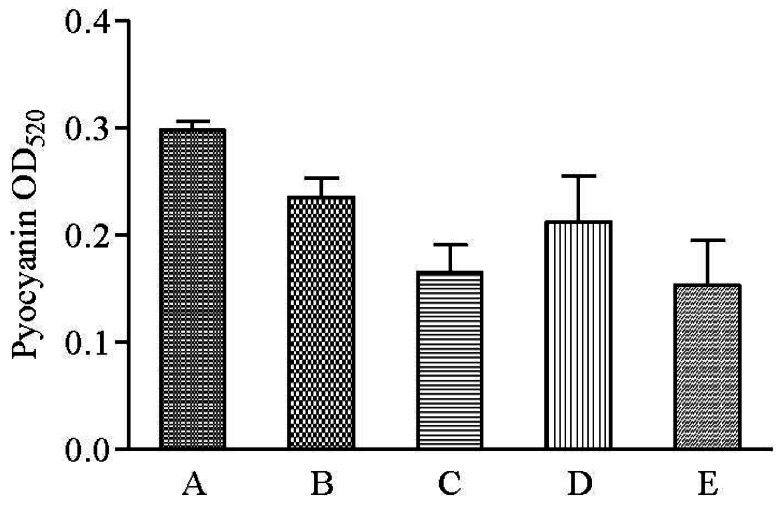
Inhibition of *P. aeruginosa* PAO1 pyocyanin production by various extracts of clove. Bar chart represents the mean of results from triplicate cultures of three independent experiments, with error bars representing standard deviations (n = 3), (**A**) *P. aeruginosa* PAO1 (no addition of solvents or extracts); (**B**) DMSO; (**C**) Hexane extract; (**D**) Chloroform extract; (**E**) Methanol extract.

QS-regulated swarming motility has been characterized as form of flagella-dependent movement on a viscous environments such as semi-solid agar surfaces while pyocyanin is characterized as a blue pigment and redox-active phenazine compound that generate reactive oxygen intermediates [28,29]. Hexane and methanolic but not chloroform extracts of clove showed reduction in pyocyanin formation (Figure 7). Both swarming and pyocyanin are produced and regulated by the *rhl* system in *P. aeruginosa* [30,31]. Therefore, collectively inhibition of *P. aeruginosa* PAO1 swarming motility and pyocyanin production suggests the presence of *rhl* inhibitor in the clove extracts.

Various extracts of clove inhibited *E. coli* [pSB401] (carries the *luxR* receptor gene) and *E. coli* [pSB1075] (carries the *lasR* receptor gene) [23] indicating the crude extracts of clove contain compounds that could serve as lead anti-QS compounds. Inhibition of PA01 *lecA::lux* fusion suggests that clove extracts might interfere with the QS-regulated *lux, rhl* and *las* systems. Clove is a traditional Ayurvedic medicinal plant, it is also widely used as spice in cuisine worldwide, and it has not been shown to exhibit adverse effects. Hence, it should be regarded as safe. However, for future work on clinical trials or application, toxicity and potential adverse effects must first be ruled out. It is suggested that cytotoxicity test should be carried out to ascertain the safety of the compounds. Taken these results together, clove extracts are shown to possess active compounds that can significantly inhibit various virulence determinants in human pathogen *P. aeruginosa* PAO1.

## 4. Conclusions/Outlook

Continuous emergence of multidrug-resistant bacteria caused increased need of anti-pathogenic and anti-infective strategy to combat bacterial infections. Natural products provide alternative medicine for treating emerging bacterial infections without leading to antibiotic resistance. Previous studies have shown anti-QS activity in plants [10,11,18,32,33]. The presence of active compounds exhibiting anti-QS activity in the clove extracts maybe useful for the development of anti-infective drugs. Our laboratory is currently elucidating the chemical structure of these active compounds to understand the anti-QS mechanism in QS bacteria.

## Figures and Tables

**Table 1.** List of strains/plasmid used.

**Strain/Plasmid**	**Description**	**Source**
**Strain**		
*C. violaceum* CV026	Mini-Tn*5* mutant derived from *C. violaceum* ATCC 31532 Hg^R^, *cvil*::Tn*5xylE*, Kan^R^, plus spontaneous Str^R^, AHL biosensor producing a purple pigment in respond to short chain AHL.	[20]
*Pseudomonas aeruginosa* strains PA01	Wild type prototroph	[21]
PA01 *lecA:lux*	*lecA::lux*CDABE genomic chromosomal reporter fusion in *P. aeruginosa* PAO1	[22]
**Biosensors**		
*Escherichia coli* [pSB401]	*luxR luxl*' (*Photobacterium fischeri* [ATCC 7744])::*luxCDABE* (*Photorhabdus luminescens* [ATCC 29999]) fusion; pACYC184-derived, Tet^R^, AHL biosensor producing bioluminescence in respond to short chain AHL	[23]
*Escherichia coli* [pSB1075]	*lasR lasl*' (*P. aeruginosa* PAO1)::*luxCDABE* (*P. luminescens* [ATCC 29999]) fusion in pUC18 Amp^R^, AHL biosensor producing bioluminescence in respond to long chain AHL	[23]
